# Uterine Hæmorrhage

**Published:** 1846-02

**Authors:** 


*Uterine Hæmorrhage at the sixth month of Pregnancy—The "Plug" and bandage applied—The Ovum Expelled in two hours after.*—March 14, 1839, half past 3 P.M., I was requested by a surgeon to visit Mrs. G——, pregnant of her seventh child, herself believing that she had arrived at the full term of her gestation. She had had trifling pains since half past 9, A.M.; the cause of the hæmorrhage unknown. On examination, judging from the form of the uterine neck, and the degree of elevation of the fundus of the uterus in the abdomen, I expressed my conviction, although assured to the contrary, that the patient had not completed the term of her pregnancy. From the softness of the presenting head, and it being only to be reached by the tip of the finger, the case had been mistaken, by a party previously consulted, for one of placenta prævia. The hæmorrhage continuing, and the orifice of the uterus being undilated, I introduced, by separate portions, a plug of soft sponge, until the vagina was completely filled; a pad was applied to the vulva, to prevent the escape of the plug, and a double-T bandage to the abdomen. The hæmorrhage was thus completely arrested. At the end of two hours, brisk expulsive action being instituted, the plug was removed, and immediately a six month's fœtus was expelled, the placenta speedily following, without difficulty. The patient's recovery was progressive and perfect. *London Lancet.*

